# Ceruloplasmin potentiates nitric oxide synthase activity and cytokine secretion in activated microglia

**DOI:** 10.1186/s12974-014-0164-9

**Published:** 2014-09-16

**Authors:** Massimo Lazzaro, Barbara Bettegazzi, Marco Barbariga, Franca Codazzi, Daniele Zacchetti, Massimo Alessio

**Affiliations:** Proteome Biochemistry Unit, San Raffaele Scientific Institute, via Olgettina 58, Milan, 20132 Italy; Cellular Neurophysiology Unit, San Raffaele Scientific Institute, via Olgettina 58, Milan, 20132 Italy

**Keywords:** Ceruloplasmin, Lipopolysaccharide, Microglial cells, Neuroinflammation, Inducible nitric oxide synthase

## Abstract

**Background:**

Ceruloplasmin is a ferroxidase expressed in the central nervous system both as soluble form in the cerebrospinal fluid (CSF) and as membrane-bound GPI-anchored isoform on astrocytes, where it plays a role in iron homeostasis and antioxidant defense. It has been proposed that ceruloplasmin is also able to activate microglial cells with ensuing nitric oxide (NO) production, thereby contributing to neuroinflammatory conditions. In light of the possible role of ceruloplasmin in neurodegenerative diseases, we were prompted to investigate how this protein could contribute to microglial activation in either its native form, as well as in its oxidized form, recently found generated in the CSF of patients with Parkinson’s and Alzheimer’s diseases.

**Methods:**

Primary rat microglial-enriched cultures were treated with either ceruloplasmin or oxidized-ceruloplasmin, alone or in combination with lipopolysaccharide (LPS). Production of NO and expression of inducible nitric oxide synthase (iNOS) were evaluated by Griess assay and Western blot analysis, respectively. The productions of the pro-inflammatory cytokine IL-6 and the chemokine MIP-1α were assessed by quantitative RT-PCR and ELISA.

**Results:**

Regardless of its oxidative status, ceruloplasmin by itself was not able to activate primary rat microglia. However, ceruloplasmin reinforced the LPS-induced microglial activation, promoting an increase of NO production, as well as the induction of IL-6 and MIP-1α. Interestingly, the ceruloplasmin-mediated effects were observed in the absence of an additional induction of iNOS expression. The evaluation of iNOS activity in primary glial cultures and *in vitro* suggested that the increased NO production induced by the combined LPS and ceruloplasmin treatment is mediated by a potentiation of the enzymatic activity.

**Conclusions:**

Ceruloplasmin potentiates iNOS activity in microglial cells activated by a pro-inflammatory stimulus, without affecting iNOS expression levels. This action might be mediated by the activation of a yet unknown Cp receptor that triggers intracellular signaling that cross-talks with the response elicited by LPS or other pro-inflammatory stimuli. Therefore, ceruloplasmin might contribute to pathological conditions in the central nervous system by exacerbating neuroinflammation.

## Background

Ceruloplasmin (Cp) is one of the major copper-binding proteins in the blood and, due to its ferroxidase activity, plays a role in iron metabolism. Cp is secreted in the plasma by the liver, but it is produced also by the epithelial cells of the choroid plexus and released in the cerebrospinal fluid (CSF) [1]. In the brain, a GPI-anchored form of Cp is present on astrocytes [2] and leptomeningeal cells [3], where it may contribute to iron homeostasis and antioxidant defense, converting toxic ferrous iron into ferric form [4,5]. We previously reported that Cp undergoes oxidative modifications in the CSF of Parkinson’s disease (PD) and Alzheimer’s disease (AD) patients [6], due to the oxidative environment of pathological CSF [7,8]. Oxidized Cp (Cp-ox) loses its ferroxidase activity, which in turn promotes intracellular iron retention in neurons [6] and gains integrin-binding and signaling properties, due to oxidation-induced structural changes [9,10].

Microglial cells are immune-competent cells derived from the monocyte/macrophage lineage and distributed throughout the central nervous system (CNS). They represent the first line of defense, being activated in response to different stimuli such as cerebral ischemia, infection, neurodegenerative disease and endotoxins [11,12]. After activation, microglial cells trigger the inflammatory processes characterized by secretion of pro-inflammatory cytokines, chemokines, elements of complement cascade and by increased expression of several enzymes responsible for the production of either reactive-oxygen or -nitrogen species (ROS and RNS) [13]. These reactive molecules are necessary at low concentrations for the defense mechanisms against invading microbial and viral pathogens, but at higher concentrations they are toxic for neurons and can accelerate and exacerbate the progression of neurodegeneration [14-18].

Several neurodegenerative disorders, including Alzheimer’s and Parkinson’s diseases, are characterized by high levels of ROS and RNS in brain, serum and CSF [18-22], suggesting that neurodegenerative disease may also be driven by an over-activation of microglial cells.

Microglial cells express Toll-like receptors (TLRs 1 to 9), responsible for the pro-inflammatory pathway activation induced by microbes, viruses and tissue damage [13,23,24]. In addition to these common stimulants, microglia can be activated by disease-specific proteins, such as β-amyloid and α-synuclein [22,25,26], and by soluble mediators released by dying neurons (for example, matrix metalloproteinase-3, calpain, neuromelanin, fractalkine) [17,27]. Microglial cells can also be stimulated by lipopolysaccharide (LPS), the principal cell-wall component of Gram-negative bacteria [23,24,28,29]. The deleterious effects of LPS might also be mediated by its interaction with TLRs present on brain endothelial cells, which, in turn, can activate adjacent microglial cells by releasing nitric oxide (NO) or other mediators [30-34]. Therefore, a possible role of LPS-mediated neuroinflammation has been proposed also in the progression of PD and AD [35-39].

It has been reported that Cp is able to activate microglia with ensuing induction of inducible nitric oxide synthase (iNOS), production of NO and increase in the levels of mRNAs encoding interleukins and enzymes such as cyclooxygenase-2 or NADPH oxidase [40]. For this reason, we were prompted to investigate whether Cp, as well as the oxidized Cp found in the CSF of PD and AD patients, could have a role in exacerbating the pro-inflammatory pathological conditions via microglial cell activation.

Here we show that Cp alone, regardless of its oxidative status, is not able to activate microglial cells, but can potentiate/synergize the LPS-induced microglial activation, increasing the production of NO, the induction of IL-6 and MIP-1α mRNAs and the secretion of IL-6. These effects were observed in the absence of further induction of iNOS expression, and sustained by the potentiation of the iNOS activity.

## Methods

### Material

Ceruloplasmin from human plasma was purchased from Enzo Life Sciences (Farmingdale, NY, USA), the presence of endotoxin contaminant was evaluated using the Limulus Amebocyte Lysate (LAL) Pyrogent® Plus Single test (Lonza, Walkersville, MD, USA) and results found to be 0.006 EU/μg of Cp. *E. coli* LPS, *N*_ω_-Nitro-L-arginine methyl ester hydrochloride (L-NAME) and other chemicals, when not specified, were obtained from Sigma-Aldrich (St Louis, MO, USA). Recombinant rat IL-1β, TNF-α, INF-γ and granulocyte macrophage colony-stimulating factor (GM-CSF) were from R&D Systems (Minneapolis, MN, USA). All the reagents were resuspended in apyrogenic endotoxin-free water for clinical injectable preparations (SALF-Laboratorio Farmacologico, Bergamo, Italy). The antibodies used in the study were mouse monoclonal anti-iNOS (BD Biosciences, San Jose, CA, USA), mouse monoclonal anti-α-tubulin (Sigma-Aldrich, St Louis, MO, USA). Polyclonal goat anti-mouse Ig horseradish peroxidase (HRP)-conjugated (DAKO, Carpinteria, CA, USA) was used as the secondary antibody.

### Cell cultures

The animal use procedures, performed according to the EC Directive 86/609/EEC, were approved by the Institutional Animal Care and Use Committee of the San Raffaele Scientific Institute. Animals were sacrificed after gentle carbonarcosis (by slowly rising CO_2_ inside the cage) to minimize pain and discomfort.

Primary rat microglial-enriched cultures were obtained from cerebral cortices of two-day-old Sprague-Dawley rat pups, as described previously [41,42]. After removing the meninges, cortices were cut into small sections and washed in Hank’s Balanced Salt Solution supplemented with Hepes/Na pH 7.4 (10 mM), MgSO_4_ (12 mM), 50 U/ml penicillin and 50 μg/ml streptomycin. Then, they were dissociated with 2.5 mg/ml trypsin type IX in the presence of 1 mg/ml deoxyribonuclease (Calbiochem, San Diego, CA, USA) for 10 minutes at 37°C in two subsequent steps and the cell suspension obtained was diluted 1:1 in medium containing 10% horse serum (PAA Laboratories, Dartmouth, MA, USA). The cell suspension was spun (50 g for 15 minutes) and cells were put in culture in Minimum Essential Medium Eagle (Lonza, Walkersville, MD, USA) supplemented with 10% horse serum, 33 mM glucose, 2 mM Glutamax (Gibco, Grand Island, NY, USA), 50 U/ml penicillin, 50 μg/ml streptomycin and 20 ng/ml GM-CSF. Cells were maintained in 75 cm^2^ flasks (approximately 1 flask for 3 pups) at 37°C in a humidified 5% CO_2_ incubator. Microglia cells were obtained by gentle manual shaking of the flasks two to three days after dissection. Detached cells (about 75 to 85% microglia with a 15 to 25% astrocytic contamination) were plated with fresh medium containing GM-CSF on 12-well plates coated with poly-l-lysine (100 μg/ml).

Purity of highly-enriched microglial cells was assessed by morphological examination performed by immunofluorescence for ionized calcium-binding adaptor molecule 1 (polyclonal rabbit anti-IBA1 (Wako, Richmond, VA, USA) used at 1:250 dilution), and glial fibrillary acidic protein (monoclonal mouse anti-GFAP (Sigma-Aldrich, St Louis, MO, USA), used at 1:250 dilution), markers for microglia and astrocytes respectively; in all the experiments purity ranged between 75 to 85%.

The resting state of unstimulated microglia was confirmed by the almost undetectable levels of IL-6 secretion and iNOS expression.

### Cp oxidation and Cp denaturation

Oxidation and deamidation of Cp, which promote its integrin-binding properties [10], were performed by incubating purified Cp at 37°C in 10 mM hydrogen peroxide solution and 100 mM ammonium bicarbonate, pH 8.5 as described in [6,10]; this product is referred in the text as oxidized-Cp (Cp-ox). Denaturation of Cp was performed by heating Cp at 100°C for 15 minutes and referred in the text as Cp-heated.

### Cell treatments

Stimuli were administered directly to the culture medium as follows: rat cortical highly-enriched microglia cultures were stimulated in serum-free fresh medium containing 33 mM glucose, 2 mM glutamine, 50 U/ml penicillin, 50 μg/ml streptomycin with: LPS (10 ng/ml), mix of IL-1β (10 ng/ml) and TNF-α (30 ng/ml) (referred in text as 2CKs), mix of IL-1β (10 ng/ml), TNF-α (30 ng/ml) and INF-γ (20 ng/ml) (referred in text as 3CKs), Cp from human plasma (1 to 20 μg/ml) and oxidized-Cp (1 to 20 μg/ml) for 24 hours at 37°C. Cytokine concentrations were used according to the literature as to obtain maximal effect on glial cells (see [43] and references therein) [41,44]. In some cases *N*_ω_-Nitro-L-arginine methyl ester hydrochloride (L-NAME), an arginine-analog that selectively inhibits iNOS function was administered at different concentrations (0.1, 0.25 and 1 mM) 1 hour before pro-inflammatory stimuli administration and left for the entire duration of the treatment.

### Nitrite assay

Nitrite present in culture supernatants was measured as an indirect indicator of NO production using the Griess assay [45]. An aliquot (100 μl) of culture medium was mixed with an equal volume of Griess reagent (1:1 mixture of 1% sulfanilamide in 5% orthophosphoric acid and 0.1% naphtylenethylenediamine dihydrochloride in H_2_O) in a 96-multiwell plate. Plates were gently shaken for 1 minute and the absorbance at 550 nm was measured using a microplate reader. Nitrite concentration in the samples was interpolated with a sodium nitrite standard curve ranging from 0 to 100 μΜ.

### Quantitative real-time PCR

RNA was extracted with TRIzol® according to manufacturer’s protocol (Invitrogen, Carlsbad, CA, USA). After extraction with chloroform and ethanol precipitation, RNA pellets were air-dried for 5 minutes, resuspended in 20 μl of RNase-free water and stored at −80°C. To evaluate the purity of RNA, the ratio of absorptions at 260 versus 280 nm (for contamination by other nucleic acids and protein) and ratio at 230 versus 260 nm (for contamination by organic compounds), were assessed with spectrophotometer (Eppendorf, Hauppauge, NY, USA). The RNA quality was assessed by electrophoresis.

Reverse transcription (RT) was performed using Superscript III Retrotranscription Kit (Invitrogen, Carlsbad, CA, USA) and random hexamers as primers, following manufacturer instruction. RT was carried out for 50 minutes at 50°C followed by incubation for 5 minutes at 85°C. Single strand cDNA was obtained digesting complementary RNA strand with provided RNase H for 20 minutes at 37°C.

Real-time comparative PCR was performed on a LightCycler® 480 Real-Time PCR System (Roche), using 500 ng of cDNAs as templates and LightCycler® 480 DNA SYBR Green I Master Mix (Roche) according to the manufacturer’s instructions. Primers were designed using Primer Express Software v3.0 (Applied Biosystems, Foster City, CA, USA) and were purchased from PRIMM (Milano, Italy). Each primer was tested to evaluate efficiency and specificity (range 91 to 103%); the RT-PCR amplification was carried out according to our previous report [41] with a denaturation step at 95°C for 10 minutes, followed by 45 cycles of amplification. Each cycle consisted of a denaturation step (95°C, 10 seconds), an annealing step (60°C, 25 seconds) and an elongation step (72°C, 15 seconds). After amplification, a melting step was performed (95°C for 30 seconds, 60°C for 1 minute). Determination of crossing points and melting peaks was performed with LightCycler 480 Software (version 1.5.0.39, Roche, Basel, Switzerland). Primers forward and reverse were used both at a 0.5 μM concentration and the sequences were: ccaccgctgcccttgctgtt and cacccggctgggagcaaagg for the gene encoding macrophage inflammatory protein-1 alpha (MIP-1α); gtatgaacagcgatgatgcact and gaagaccagagcagattttcaatag for the gene encoding IL-6; cagaaggacgtgaaggatgg and cagtggtcttggtgtgctga for 18S rRNA, that is the gene coding for 18S ribosomal RNA, used as internal reference for normalization.

Each sample was analyzed in duplicate. For each sample, relative expression of target genes was calculated based on real-time PCR efficiencies (*E*) and the threshold cycle (Ct) difference (Δ) of a treated sample versus a control (ΔCt_control–sample_) and expressed relative to the reference genes chosen, in according to the 2^–ΔΔCt^ method.

### Western blot analysis

Cells were lysed on ice in lysis buffer (15 mM PBS, 2% NP-40, 0.2% SDS, 10 mM EDTA, 1% protease inhibitor cocktail, 1% phosphatase inhibitor cocktail). After centrifugation, the supernatant was collected and protein concentration evaluated by Bradford assay (Bio-Rad Laboratories, Hercules, CA, USA). Lysates containing 10 to 20 μg of proteins were re-suspended in Laemmli buffer, then proteins were resolved on 10% acrylamide SDS-polyacrylamide gel electrophoresis and then electro-transferred to nitrocellulose membranes for Western blot (WB) analysis. Protein transfer was evaluated by red Ponceau S staining (Sigma-Aldrich, St Louis, CA, USA). The membranes were blocked in a 5% milk solution in TBS (0.1% Tween 20) and incubated 12 hours at 4°C with primary antibodies. The reactivity was revealed by incubation (1 hour at 20°C) with HRP-conjugated secondary rabbit anti-goat IgG followed by chemiluminescence reaction performed with electrochemiluminescence (ECL) detection reagents (GE Healthcare, Little Chalfont, UK) and film exposure. The WB bands reactivities were quantified by densitometry analysis using a G-Box scanner and the associated GeneSys software (Syngene, Cambridge, UK). The films were scanned and the bands optical density was measured with GeneTools software (Syngene, Cambridge, UK). Expression of α-tubulin was used as a loading control.

### Interleukin-6 determination

IL-6 was measured by ELISA kit (KRC0061; Invitrogen, Carlsbad, CA, USA), according to manufacturer’s instructions, using 50 μl of supernatants collected from microglial cultures after different treatments.

### Nitric oxide synthase activity

NOS activity was evaluated with the Colorimetric Nitric Oxide Assay Kit from Oxford Biomedical Research (Oxford, UK). The assay measures the capability of NOS, present in cell lysates, to convert L-arginine to citrulline and NO and with a colorimetric reaction reveals nitrite and nitrate production as read-out products. For the assay, 100 μg of proteins were used from primary rat microglial-enriched cultures treated for 24 hours with LPS 10 ng/ml, alone or in combination with Cp 20 μg/ml. Cells were lysed on ice in PBS containing 0.5% Triton X-100, 1% protease inhibitor cocktail, 1% phosphatase inhibitor cocktail. After centrifugation, the lysate supernatant was collected, protein concentrations determined by Bradford assay (Bio-Rad Laboratories, Hercules, CA, USA), and lysates were used for activity detection.

### Statistical analysis

Continuous data were evaluated by Mann-Whitney test, since they did not pass the normality test for Gaussian distribution, as assessed by the Kolmogorov-Smirnov test; two-tailed *P*-value was used for the comparison of two means and standard error; the mean values were calculated using pooled data from different experiments. In all analyses, *P* < 0.05 was considered to be statistically significant. The analysis was performed with Prism V4.03 software (GraphPad Inc., La Jolla, CA, USA).

## Results

### Cp and Cp-ox potentiate LPS-induced NO production in the absence of additional iNOS induction

**Figure 1:**
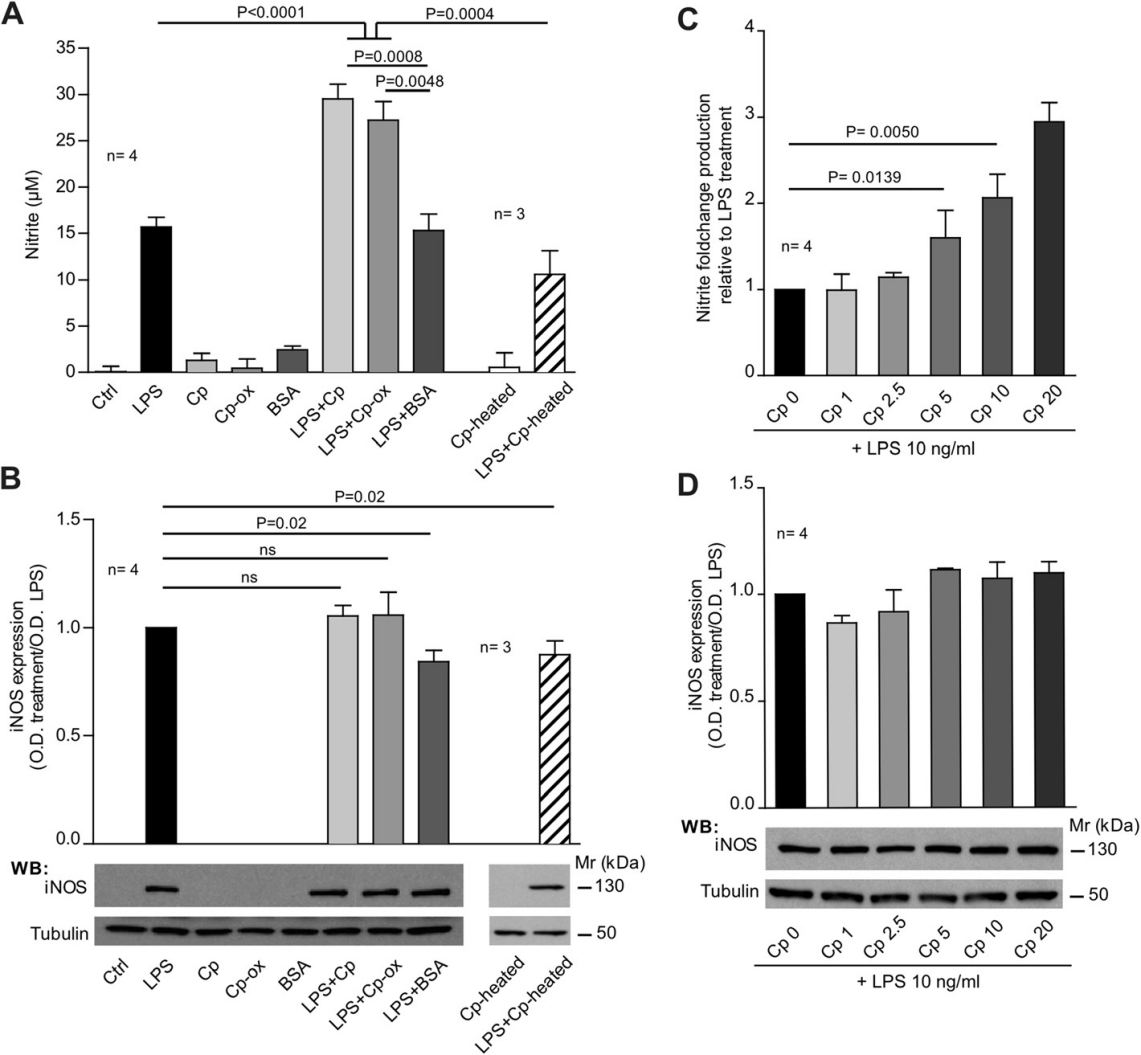
**Cp and Cp-ox potentiate lipopolysaccharide (LPS)-induced nitric oxide (NO) production in the absence of additional inducible nitric oxide synthase (iNOS) induction. (A)** Nitrite production assessed by Griess assay in culture medium of microglial cells after 24 hours of treatment with medium alone (Ctrl), LPS (10 ng/ml), ceruloplasmin (Cp), oxidized-ceruloplasmin (Cp-ox), heat-denatured ceruloplasmin (Cp-heated) and BSA alone (all at 20 μg/ml) or in combined treatment with LPS. Results are expressed as μM of nitrite present in culture medium that reflects the NO-production. **(B)** Western blot analysis of inducible nitric oxide synthase expression in microglial cells after treatments as described in **(A)**. Densitometric optical density (OD) for iNOS bands were normalized with α-tubulin expression and are reported as ratio of the OD of specific treatments versus OD of LPS treatment. Bottom panels are representative of one experiment. **(C)** Dose-dependent analysis of Cp co-treatment in the potentiation of the LPS-induced nitrite production. Microglial cells were treated with a steady amount of LPS (10 ng/ml) plus increasing concentrations of Cp (1, 2.5, 5, 10 and 20 μg/ml). The nitrite production was reported as ratio of nitrite production in specific treatment versus LPS treatment. **(D)** Western blot analysis for iNOS expression in microglial cells upon LPS treatment combined with dose-dependent increase of Cp. Densitometric analysis was reported as described in (B). Bottom panels are representative of one experiment. Three/four independent experiments (as indicated n =) were performed and mean values, calculated using pooled data from different experiments, with standard error are reported. Statistical *P*-values were evaluated by non-parametric Mann-Whitney test. In all analyses, *P* < 0.05 was considered to be statistically significant.

Primary rat microglia-enriched cultures treated with LPS at 10 ng/ml showed an activated phenotype, as expected, and displayed both iNOS expression and nitrite increase (15.9 ± 2 μM) in the medium (Figure 1A-B). On the contrary, treatment of cells with either Cp, oxidized-Cp or with the control BSA stimulus (20 μg/ml), did not trigger activation, as assessed by nitrite dosage in the cell medium (Figure 1A). As expected, the same treatments did not induce iNOS expression as evaluated by WB (Figure 1B). Interestingly, the concomitant treatment of LPS with Cp or with Cp-ox produced a synergistic effect that significantly increased (*P* < 0.0001, Mann-Whitney test) the amount of nitrite detected in the medium (30 ± 2 μM), even in the absence of a further induction of the iNOS enzyme (Figure 1A-B). A similar synergistic effect was not observed if microglial cells were concomitantly treated with LPS and BSA (Figure 1A-B).

The concomitant administration of LPS and heat-denatured Cp to microglial cells indicated that the observed potentiating effect of Cp was due to Cp protein features. Indeed the loss of Cp protein conformation resulted in a significant failure (*P* = 0.0004, Mann-Whitney test) of the synergistic effect with LPS (Figure 1A-B).

Analysis of the synergy between Cp and LPS on activation showed a dose-dependent increase of nitrite production, evaluated in rat primary microglial cells treated with 10 ng/ml LPS plus increasing concentration of Cp (0, 1, 2.5, 5, 10, 20 μg/ml). Results showed a significant increase in the nitrite production already at 5 μg/ml Cp (*P* = 0.013, Mann-Whitney test), a concentration that is consistent with the physiological Cp concentration in the CSF (Figure 1C). Also in this case, WB analysis showed no significant differences in iNOS expression in cells treated with different Cp concentrations in addition to LPS (Figure 1D).

To confirm that the observed effects were attributable to microglial cells activation and not to the few astrocytes present in the cultures, we performed LPS and LPS + Cp treatments on primary astrocytes cultures, showing neither production of nitrite nor iNOS expression (data not shown).

### Cp and Cp-ox strengthen cytokines production in LPS-induced microglial activation

**Figure 2:**
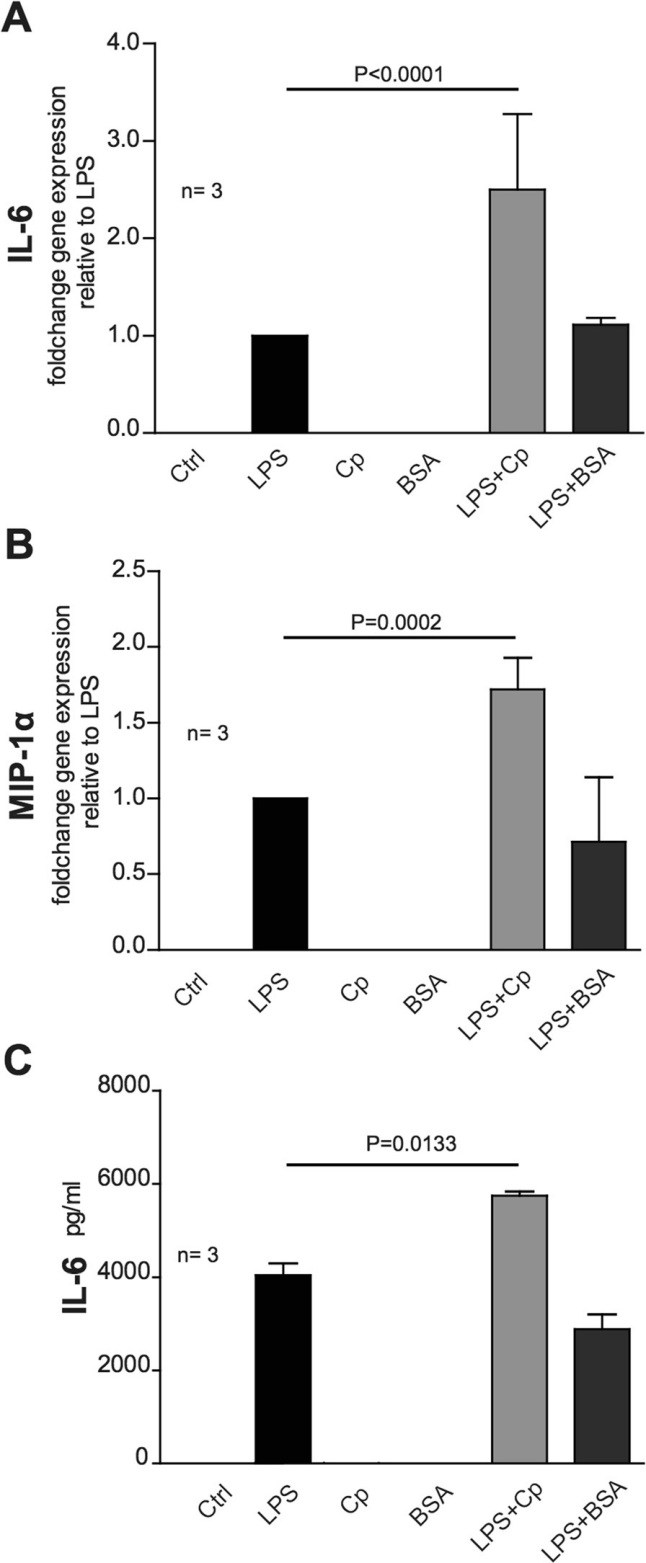
**Cp increases cytokines production in lipopolysaccharide (LPS)-induced microglial activation. (A)** Expression of IL-6 cytokine mRNA assessed by quantitative-PCR in microglial cells after 24 hours of treatment with medium alone (Ctrl), LPS (10 ng/ml), ceruloplasmin (Cp), and BSA alone (20 μg/ml) or in combined treatment with LPS. Results are expressed as mRNA fold change expression levels relative to expression value obtained for LPS stimulus. **(B)** Expression of MIP-1α chemokine mRNA assessed by quantitative PCR in microglial cells as in **(A)**. Results are expressed as mRNA fold change expression levels relative to expression value obtained for LPS stimulus. **(C)** Secretion of IL-6 cytokine was evaluated by ELISA test in the culture medium of microglial cells after treatments as in **(A)**. Results are reported as picograms of protein per milliliter of medium. Three independent experiments (n = 3) were performed and mean values, calculated using pooled data from different experiments, with standard error are reported. Statistical *P*-values were evaluated by non-parametric Mann-Whitney test. In all analyses, *P* < 0.05 was considered to be statistically significant.

In order to assess whether NO production was associated with a general activation of microglial cells, we tested, by real-time PCR, the expression of the prototypic pro-inflammatory cytokine IL-6 and chemokine MIP-1α. LPS treatment induced the expression of these genes, while neither Cp alone nor BSA treatments resulted to be effective (Figure 2A-B). Similar to what we observed for NO production, co-treatment with LPS and Cp showed a synergistic effect, further increasing the expression of both IL-6 and MIP-1α (Figure 2A-B).

The synergistic effect of Cp co-treatment in the induction of IL-6 cytokine expression was also confirmed at protein level by ELISA test. Indeed, Cp caused a significant increase in the amount of secreted IL-6 in the culture supernatants in comparison to LPS alone or LPS + BSA (Figure 2C).

No differences were observed between Cp and Cp-ox treatment (data not shown), thus definitively ruling out the initial hypothesis of a possible gain of different function in microglial stimulation by Cp-ox.

### The synergistic effect of Cp in microglia activation depends on the presence of iNOS

**Figure 3:**
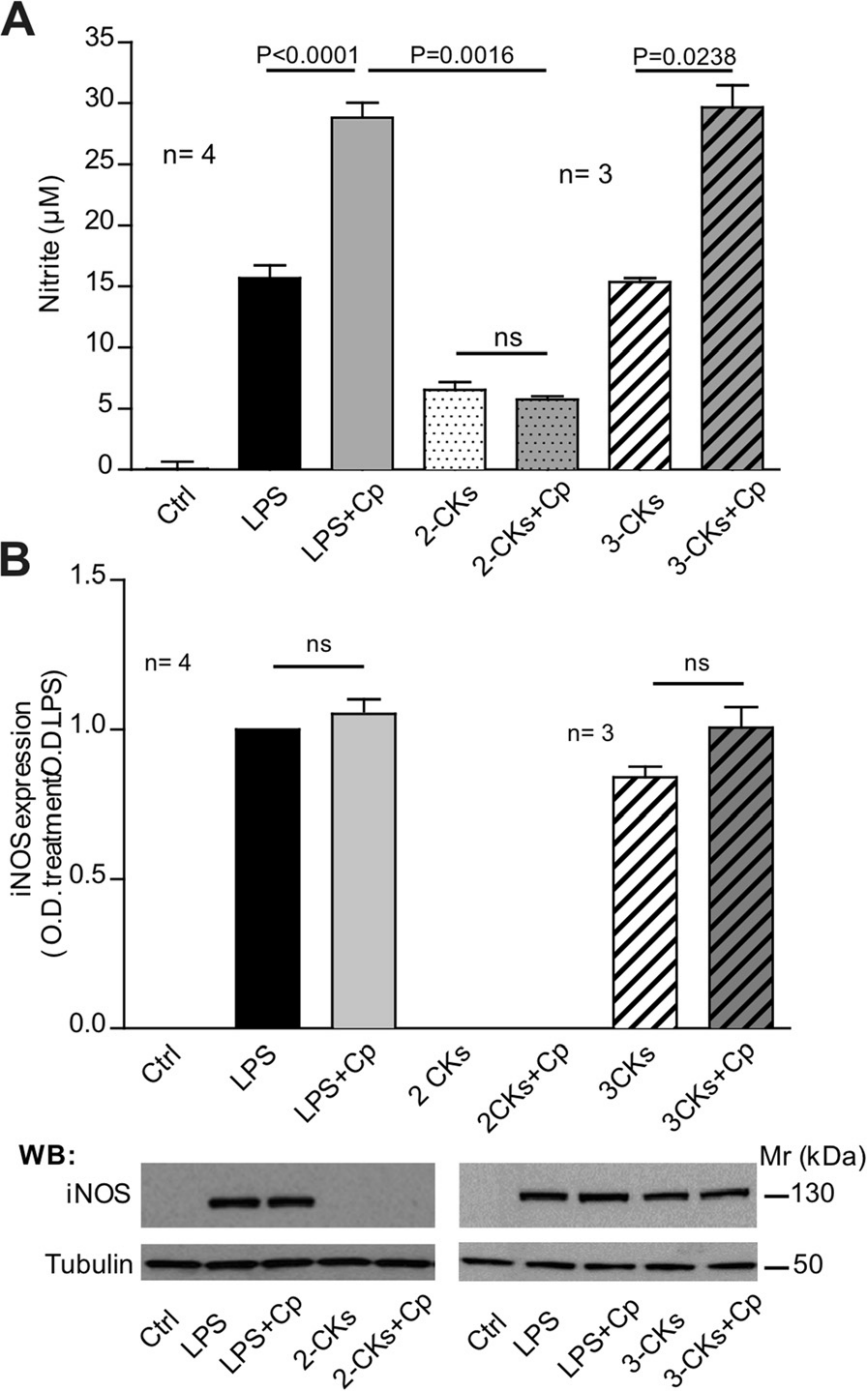
**The effect of Cp in microglial activation depends on the presence of inducible nitric oxide synthase (iNOS). (A)** Nitrite production assessed by Griess assay in culture medium of microglial cells after 24 hours of treatment with medium alone (Ctrl), LPS (10 ng/ml), IL-1β and TNF-α (2-CKs), or with IL-1β, TNF-α and IFN-γ (3-CKs) alone or in combination with ceruloplasmin (Cp) (20 μg/ml). Results are expressed as μM of nitrite present in culture medium that reflects the nitric oxide (NO)-production. **(B)** Western blot analysis of iNOS expression in microglial cells after treatments as in **(A)**. Densitometric optical density (OD) for iNOS bands were normalized with α-tubulin expression and are reported as ratio of the OD of specific treatments versus OD of LPS treatment. Bottom panels are representative of one experiment. Three/four independent experiments (as indicated n =) were performed and mean values, calculated using pooled data from different experiments, with standard error are reported. Statistical *P-*values were evaluated by non-parametric Mann-Whitney test. In all analyses, *P* < 0.05 was considered to be statistically significant.

Induction of iNOS protein in microglia occurs in response to LPS, but microglia can also be activated by other stimuli. To investigate whether the observed synergistic effect of Cp was specific for LPS activation, we concomitantly treated with Cp primary microglia cultures stimulated with mix of different cytokines (CKs), IL-1β and TNF-α (herein after referred to as ‘2-CKs’), or with IL-1β, TNF-α and IFN-γ (hereinafter referred to as ‘3-CKs’) known to be respectively unable and able to induce iNOS expression [46]. The use of 2-CKs alone showed very low nitrite production with respect to LPS stimulation, and the results were similar when 2-CKs were used in combination with Cp (Figure 3A); in both conditions iNOS protein expression was not detectable (Figure 3B). On the contrary, the treatment with 3-CKs resulted in a nitrite production comparable to those noticed in microglia stimulated with LPS, and when 3-CKs were supplemented with Cp, a synergistic effect on nitrite production, comparable to that detected with LPS + Cp, was observed (Figure 3A). As expected, the use of IFN-γ together with IL-1β and TNF-α induced the expression of iNOS, and, similarly to the LPS + Cp treatment, no further expression changes were induced by concomitant treatment with Cp (Figure 3B). mRNA expression of both IL-6 and MIP-1α, and the release of IL-6 protein in the medium, was found to be weak after microglia were incubated with either 2-CKs or 3-CKs, and were not modified by the addition of Cp (data not shown).

### Increased NO production fostered by LPS + Cp co-treatment depends on incremented iNOS activity

In order to investigate whether increased NO production induced by Cp co-treatment was dependent on the modulation of iNOS activity, we utilized L-NAME, an arginine-analog that selectively inhibits iNOS function.

**Figure 4:**
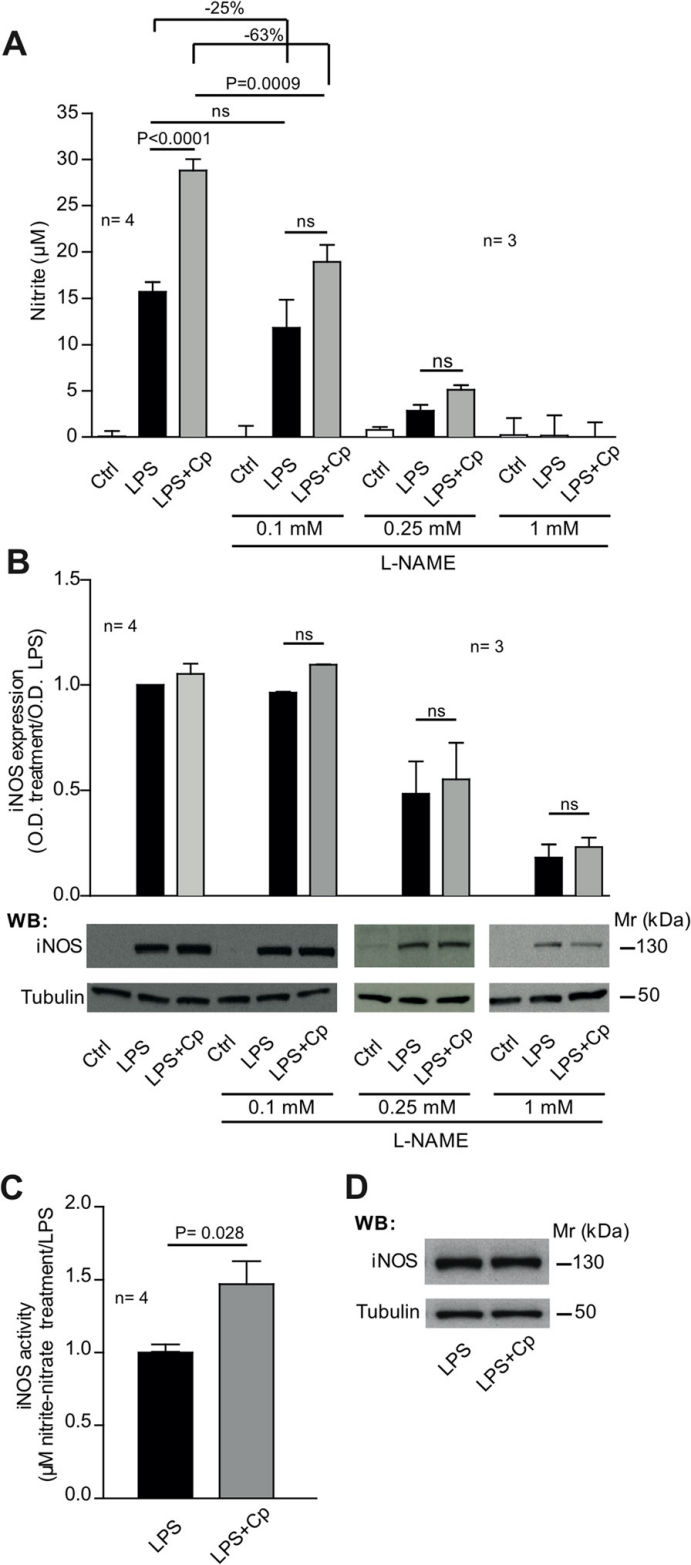
**Increased nitric oxide (NO) production fostered by Cp treatment in combination with lipopolysaccharide (LPS) depends on an incremented inducible nitric oxide synthase (iNOS) activity. (A)** Nitrite production assessed by Griess assay in culture medium of microglial cells after 24 hours of treatment with medium (Ctrl), LPS (10 ng/ml) alone and in combination with Cp (20 μg/ml) or the same treatments performed following 1 hour pre-treatment with increasing concentrations of L-NAME (0.1, 0.25 and 1 mM). Results are expressed as μM of nitrite present in culture medium that reflects the NO production. Reduction percentage of nitrite production induced by L-NAME pre-treatment is indicated. **(B)** Western blot analysis of iNOS expression in microglial cells after treatments as in **(A)**. Densitometric optical density (OD) for iNOS bands was normalized with α-tubulin expression and was reported as ratio of the OD of specific treatments versus OD of LPS treatment. Bottom panels are representative of one experiment. **(C)** Measure of iNOS activity in the lysate of microglial cells after LPS and LPS + Cp treatment. Results are reported as the ratio of nitrite and nitrate production (μM) in the lysate of cells treated with LPS + Cp versus LPS alone. Nitrite production was normalized by iNOS and α-tubulin expression levels as revealed by Western blot densitometric analysis. **(D)** Representative Western blot analysis of iNOS and α-tubulin expression in lysates of microglial cells treated with LPS and LPS + Cp that were used for enzyme activity normalization. Three/four independent experiments were performed (as indicated n =) and mean values, calculated using pooled data from different experiments, with standard error are reported. Statistical *P*-values were evaluated by non-parametric Mann-Whitney test. In all analyses, *P* < 0.05 was considered to be statistically significant.

We found that at low concentration (0.1 mM) L-NAME produced only a small reduction in the amount of NO produced by LPS stimulation (25% reduction compared to cells treated with LPS alone, not statistically significant). On the contrary, L-NAME pre-treatment was more effective in reducing NO production in cells concomitantly treated with LPS and Cp (63% reduction of the increased NO production over LPS treatment alone, *P* = 0.0009, Mann-Whitney test), almost abolishing the synergistic effect (Figure 4A).

In both LPS and LPS + Cp treatments, in the presence of L-NAME 0.1 mM, iNOS protein expression levels remained equal to those observed in the absence of the iNOS inhibitor (Figure 4B). These results together suggested that the Cp-induced synergistic effect on NO production could depend on the modulation of iNOS activity. Increasing L-NAME concentration to 0.25 mM caused a strong decrease in NO production in both LPS and LPS + Cp treatments, resulting also in a reduction in iNOS protein expression of about 50%, as determined by WB analysis (Figure 4A-B). At higher L-NAME concentration (1 mM), the nitrite production was completely abolished, and the iNOS protein expression was reduced of about 75%, if compared with LPS treatment (Figure 4A-B).

To confirm that the increase of nitrite production observed in LPS + Cp treatment was dependent on an increase in iNOS enzymatic activity, we measured *in vitro* the NOS activity in lysates obtained from microglial cells treated either with LPS alone or LPS + Cp. The measured activity was normalized by iNOS and α-tubulin expression, detected by WB analysis. The results showed a significant increase of about 50% (*P* < 0.0284, Mann-Whitney test) in nitrite and nitrate production by iNOS enzyme present in the lysate of microglial cells treated with LPS + Cp compared to cells treated with LPS alone (Figure 4C), despite the presence of equal iNOS expression levels, as evidenced by WB analysis (Figure 4D).

## Discussion

Here we show how microglial activation, following either LPS or cytokines mixture (IL-1β, TNF-α and IFN-γ) stimulation, is exacerbated by the concomitant treatment with ceruloplasmin, suggesting that this protein can act as a co-factor in the inflammation process. Although its role in inflammation is not completely understood, Cp has been reported to be an acute phase protein with anti-inflammatory properties, because the Cp levels increased during inflammation/infection [47,48]. Since Cp interacts with metal ions, namely copper and iron, it is very susceptible to redox changes. Recent work of our team showed that Cp oxidation, which occurs in neurodegenerative diseases as a consequence of an oxidative environment, promotes the gain of integrin-binding function and triggers intracellular signaling, that through ERK1/2, Akt and mitogen-activated protein kinase (MAPK) signaling pathways involvement, may regulate gene activation, cell cycle and proliferation [6,10].

Since it has been reported that Cp can be involved in microglial activation [40], and considering that microglial cells could play a role in many inflammatory and neurodegenerative processes in the CNS, we investigated whether Cp and Cp-ox had a role in microglia-mediated inflammatory reaction. Our results show that Cp potentiated microglial activation, promoting a significant increase in NO production. This effect was dependent on the expression of iNOS induced by a pro-inflammatory stimulus (for example, LPS or cytokines), in contrast to what was previously reported [40]. In fact, in our experimental conditions, Cp alone was not able to induce microglial activation and NO production. This contrasting observation might depend on various experimental variables such as the source of purified Cp, as well as the activation state of microglial cells, depending on culture conditions before the stimulation. In order to investigate glial activation *in vitro*, microglial cells must be in a ‘resting’ state (that is almost undetectable levels of basal secretion of pro-inflammatory molecules) until exposed to stimuli. Our culture conditions (for example, the use of horse serum, the timing of the shaking procedure, the addition of GM-CSF just after the dissection procedure, and so on) have been optimized in order to allow proliferation without inducing ‘basal’ activation in the absence of stimulation. This allowed us to better mimic the *in vivo* situation.

The findings that the oxidation status of Cp has no measurable effect on the ability of Cp to potentiate iNOS activity, rule out the initial hypothesis that Cp-ox might have a role in neuroinflammation in neurodegenerative diseases acting differently and directly on microglia. Nevertheless, a contribution to neuroinflammation in neurodegenerative diseases of Cp-ox, that has been reported to be on average about 50% of the total Cp compared to the 20% in healthy subjects [6], could be indirectly exerted throughout the release, upon oxidation, of the six copper ions coordinated in Cp structure [1,6,10,49]. Of note, the potentiation of LPS-induced NO production supported by a second stimulus has already been described in microglia in the case of the exposure to metals such as zinc, manganese and cobalt. However, in these studies, the increase in NO production was due to a concomitant increase in iNOS expression [50-53]. Our results indicate that the effect of Cp on NO production did not rely on an additional increase of iNOS expression, but rather on a potentiation of iNOS enzymatic activity. Moreover, the downstream signaling activated by Cp, not only accomplished the potentiation effect of iNOS activity but, eventually, fostered the induction of IL-6 and MIP-1α expression.

An open question that needs further investigation is how Cp mediates the potentiation of iNOS activity; one possibility, is that Cp, activating an unknown receptor, triggers an intracellular signaling that interacts with the response elicited by LPS or other pro-inflammatory stimuli. The involvement of p44/42 MAPK kinases (ERK1/2) has been reported in Cp-mediated induction of iNOS in microglial cells [40] and it is supported also by our preliminary results (data not shown); these kinases might also mediate the iNOS activity potentiation induced by Cp co-stimulation. In fact, the ERK-mediated phosphorylation of human iNOS on Serine 745 (rat ortholog Ser742) has been reported to be a stimulator of iNOS enzymatic activity [54].

Although neuroinflammation is not considered as an initiating factor in neurodegeneration, evidence obtained from animal models supports the hypothesis that inflammatory responses involving microglia contribute to neurodegenerative diseases progression [14,15,22,23,25,37]. We used LPS as a paradigm for microglial activation that is usually due to disease-specific proteins and soluble mediators. *In vivo* LPS can trigger microglial activation either directly, entering the CNS through a damaged blood-brain-barrier (BBB) [24,32,55,56], or indirectly through molecules released by endothelial cells upon interaction with bacteria [30,31,33].

If the Cp-mediated reinforcement of microglial activation occurs in brain, the increased production of neurotoxic compounds like NO might contribute to neurodegeneration, since NO can react with free radical superoxide to form peroxynitrite, a powerful oxidizing agent with potent cytotoxic action [57,58]. The physiological Cp concentration in CSF is on average 1.5 μg/ml [59], which is lower than the concentration we found efficacious in producing the potentiation effect (5 to 20 μg/ml); nevertheless, Cp concentration can locally increase, as it occurs in some brain regions after injury or in neurodegenerative disorders [60-62]. In addition, it must be taken into consideration that BBB damage could allow both serum penetration and infiltration of white blood cells, that could further increase local Cp concentration. In fact, Cp concentration in serum is ten fold higher than in the CSF, and it has been reported that lymphocytes and monocytes/macrophages express both the soluble and the GPI-anchored Cp isoforms [63-65]. The local increase of Cp concentration due to serum Cp penetration might also be fostered by the release of copper ions from the Cp-ox in the neurodegenerative CSF, which in turn could affect the physiological functions of the brain barrier systems, contributing to the blood-cerebrospinal fluid-barrier (BCB) and BBB leakiness found in some neurodegenerative disorders [66,67].

## Conclusion

Our results suggest that endogenous Cp, which usually plays an anti-inflammatory/antioxidant role, if present in increased concentration could exacerbate the damaging effect of pro-inflammatory stimuli in brain by modulating microglial activation.

## Abbreviations

AD: Alzheimer’s disease
BBB: blood-brain barrier
CK: cytokines
CNS: central nervous system
Cp: ceruloplasmin
Cp-ox: oxidized ceruloplasmin
CSF: cerebrospinal fluid
ECL: electrochemiluminescence
ELISA: enzyme-linked immunosorbent assay
GFAP: glial fibrillary acidic protein
GM-CSF: granulocyte macrophage colony-stimulating factor
HRP: horseradish peroxidase
Ig: immunoglobulin
IL: interleukin
INF-γ: interferon γ
iNOS: inducible nitric oxide synthase
L-NAME: *N*_ω_-Nitro-L-arginine methyl ester hydrochloride
LPS: lipopolysaccharides
MAPK: mitogen-activated protein kinase
MIP-1α: macrophage inflammatory protein 1α
NO: nitric oxide
PD: Parkinson’s disease
qRT-PCR: quantitative real-time polymerase chain reaction
ROS: reactive-oxygen species
RNS: reactive-nitrogen species
RT: reverse transcription
TLRs: Toll-like receptors
TNF-α: tumor necrosis factor α
WB: Western blot
